# Why Pakistani Medical Graduates Must Remain Free to Emigrate

**DOI:** 10.1371/journal.pmed.0050002

**Published:** 2008-01-29

**Authors:** Zarmeneh Aly, Fawad Taj

## Abstract

What is forgotten in the debate on brain drain, say the authors, is that some doctors who emigrate to the West have every intention of returning after their higher-level training overseas.

The current debate about the brain drain of health professionals from low-income countries such as Pakistan to the rich world often demonizes medical graduates who choose to leave their countries. Such graduates are sometimes considered to be insensitive to the plight of their country's struggling health sector. But what is forgotten in this debate is that some doctors who emigrate to the West have every intention of returning after their higher-level training overseas. And while the brain drain is often blamed for Pakistan's difficulty in meeting its people's health care needs, other factors play a major role, including the increasing demand for health care from the growing population and the adverse conditions that cause disease [1].

There are no reliable statistics on the total numbers of students from low-income countries who receive higher-level training abroad, nor on what proportion of these students return to their home countries [2]. It is true that large numbers of medical graduates leave Pakistan. A 2005 study by Fitzhugh Mullan found that Pakistan had contributed about 13,000 medical graduates to the United States, the United Kingdom, Canada, and Australia [3]. But a proportion of these Pakistani graduates do return. For example, by 2004, Pakistan's Aga Khan University had produced 1,100 graduates, 900 of whom went on to higher-level training in the US—but about 40 alumni have so far returned [4].

## Why Do Medical Graduates Leave?

There are four main reasons why medical graduates of Pakistan emigrate to the West [5]:

1. The long-standing belief of young doctors and their parents that training outside their home country is superior and a mark of achievement.
2. The expectation of bigger incomes.
3. The lure of high-tech training and super-specialization.
4. A reaction against the Pakistani government's poor management of the education system, and the corruption associated with this management, in favor of what are perceived to be the more merit-based medical training systems of the West.

Some doctors who emigrate to the West have every intention of returning.

Medical students plan their migration well in advance: most start planning to take the US medical licensing examination or the PLAB (the Professional and Linguistic Assessments Board) test immediately after graduation, or sometimes even sooner. Such advanced preparation is a marker of our foresightedness, and not simply an indication of our desperation to leave. The undergraduate medical students of the Indian subcontinent are a motivated generation. The world's rapid progress excites us, and we want to be involved in the latest medical advances. Very few graduates would be able to pursue their ambition of training in, for example, interventional radiology or developmental pediatrics while staying in Pakistan. While it is true that postgraduate training in the United States is expensive (graduate training in the US costs up to US$20,000 [4]), such costs are seen as a wise investment.

There is also evidence that some low-income countries intend for some of their medical graduates to work abroad. For example, in their study of international physician migration, Mélanie Bourassa Forcier and colleagues noted that some low-income countries, such as Cuba, India, and the Philippines, systematically train more physicians than they need, sending them abroad to benefit from remittances that are crucial to the long-term economic development of the home country [6].

## Migration Should Not Be Banned

There have been repeated calls to restrict medical graduates from migrating from poor countries. Delanyo Dovlo, for example, a specialist in human resources for health based in Accra, Ghana, suggested that “moral arguments” must be used to “create policies that moderate the loss of trained health workers from poor countries and stop the medical training subsidies they make to rich countries” [7]. But the right of individuals to leave their country, and conversely their right not to be forced to leave, are generally recognized tenets of international law [8]. And forcing people against their will to serve in an environment that is unsuitable for them will drastically hinder their job satisfaction and performance. The challenge is to advance human health while protecting health workers' rights to seek gainful employment [9].

Medical graduates tend to concentrate themselves in urban capitals where they earn more than those working in rural areas, and banning migration would not redress this imbalance. To reduce migration, local working conditions must be addressed, instead of expecting graduates to lower their aspirations and standards. The first responsibility for action belongs with each country to “train, retain, and sustain” its workforce through implementing national plans that improve salaries and working conditions; revitalizing education; and mobilizing paraprofessional and community workers, whose services are demonstrably more cost-effective and who are less likely than physicians to emigrate [10]. There is also anecdotal evidence that increasing the doctor–population ratio by expanding medical school intake and by creating new teaching hospitals may help to retain medical graduates [11]. Alternatives to expanding medical school intake might include changing the skill mix of care delivery, substituting other health professionals, or increasing productivity of health care teams.

Forcier and colleagues argue that since the international migration of physicians is likely to continue, the focus should be on “how the process of international migration of physicians can be managed and regulated in ways that confer benefits on both home and host countries” [5]. Box 1 summarizes their key suggestions. The Health Worker Migration Policy Initiative, under the leadership of Mary Robinson, President of Realizing Rights (http://www.realizingrights.org/), is aimed at finding practical solutions to the worsening problem of health worker migration from developing to developed countries. One of the initiative's first priorities will be to support the World Health Organization in drafting a framework for an International Code of Practice on Health Worker Migration [12], as called for by a resolution (WHA57.19) of the World Health Assembly in 2004 [13]. This framework will promote ethical recruitment, the protection of migrant health workers' rights, and remedies for addressing the economic and social impact of health worker migration in developing countries. One can only hope that these remedies herald a new era in which students of developing countries are empowered to serve their country while at the same time pursuing their ambitions.

**Box 1. Forcier and Colleagues' Key Suggestions to Home and Host Countries on Managing and Regulating International Physician Migration**

Home Countries

- Should face the reality of the globalization of the workforce and allow freedom of movement
- May benefit from financial remittances sent home, and from the advanced skills of physicians who return to the home country
- Can retain physicians by improving pay, working conditions, and educational opportunities
- Can offer incentives to physicians to return home, such as guaranteed employment

Host Countries

- Need to adopt codes of good practice on international physician migration
- Should consider compensation to home countries for the “cost of educating and training physicians and the value of health care services that would have been provided if the physician had not emigrated”
- Can help low-income countries in sustaining a domestic supply of physicians by strengthening development aid policies (e.g., setting up projects of shared learning with home countries or supporting transfer of medical technology to home countries)

Adapted from [6].

## Supporting Repatriation

The marked underinvestment in health at the national and state levels in Pakistan contributes to poor staffing and morale at government hospitals and clinics. Increased investment and modernization initiatives would create opportunities for physicians to work in their home country, promoting the repatriation of Pakistani medical graduates who undergo higher-level training overseas.

Saad Shafqat and Anita Zaidi of Aga Khan University Medical College have argued that the US medical community should help to support such repatriation: “One approach is to offer motivated IMGs [international medical graduates] mentoring to equip them with skills needed in their home countries. The scheme could be formalized through international cross-appointments for mentor and mentee at each other's institutions and a bilaterally recognized role for the mentor. Such initiatives are desperately needed; properly done, repatriation of IMGs can help diminish vast disparities in health care” [4].

The authors note that once US-trained physicians are reabsorbed back into their homeland, they often prove to be leaders in their area of expertise and help to build better functioning health care systems. In addition, they promote an environment conducive to research, which in turn is responsible for the attraction of a large amount of international investments in the form of funds and grants, both to support research and educational programs. Repatriated Aga Khan graduates, for example, have won grants from major international agencies, established nonprofit research organizations, joined hospitals serving refugee populations, and led disease control programs.

## Conclusion

As medical students, our proposition to return to our homeland once we have satisfied our ambitions abroad is often met with mockery and outright disbelief by both friends and relatives. It is hard for them to understand why, once we are used to the environment of the developed world, we would return to share the benefits of our experiences. No one wants to give up the comfort of home and loved ones in exchange for living amongst strangers. But we realize that staying might make us susceptible to the mundane routine, devoid of intellectual stimulus, that comprises most of the postgraduates' lives here. We realize that we'll come back better equipped with the technical know-how and academic expertise synonymous with postgraduate training in the West, with new ideas and challenges, and with the immense task of adapting our expertise to the local environment of our homeland.

## References

[pmed-0050002-b001] Talati JJM, Pappas G (2006). Migration, medical education, and health care: a view from Pakistan. Acad Med.

[pmed-0050002-b002] Rosenzweig MR (2007). Higher education and development. http://siteresources.worldbank.org/INTABCDE2007BEI/Resources/MarkRosenzweigConferenceVersion.pdf.

[pmed-0050002-b003] Mullan F (2005). The metrics of the physician brain drain. N Engl J Med.

[pmed-0050002-b004] Shafqat S, Zaidi AK (2007). Pakistani physicians and the repatriation equation. N Engl J Med.

[pmed-0050002-b005] Goyal SK (2005). Understanding the medic brain drain. Student BMJ.

[pmed-0050002-b006] Forcier MB, Simoens S, Giuffrida A (2004). Impact, regulation and health policy implications of physician migration in OECD countries. Hum Resour Health.

[pmed-0050002-b007] Dovlo D (2005). Taking more than a fair share? The migration of health professionals from poor to rich countries. PLoS Med.

[pmed-0050002-b008] Dowty A (1986). Emigration and expulsion in the Third World. Third World Q.

[pmed-0050002-b009] Daviratanasilpa S, Sriaroon C, Loghmanee D, Wilde H, Sitprija V (2003). Medical residency training in the US: important considerations. J Med Assoc Thai.

[pmed-0050002-b010] National Programme for Family Planning and Primary Health Care (2007). “The Lady Health Workers Programme”: 2003–2008. Government of Pakistan, Ministry of Health. http://www.phc.gov.pk/Downloads/PC-1%20aug.pdf.

[pmed-0050002-b011] Bloor K, Hendry V, Maynard A (2006). Do we need more doctors. J R Soc Med.

[pmed-0050002-b012] World Health Organization (2007). New initiative seeks practical solutions to tackle health worker migration. http://www.who.int/mediacentre/news/notes/2007/np23/en/index.html.

[pmed-0050002-b013] World Health Organization (2004). World Health Assembly raises global public health to new level. http://www.who.int/mediacentre/news/releases/2004/wha4/en/.

